# Laboratory Evaluation of the Properties of Asphalt Mixture with Wood Ash Filler

**DOI:** 10.3390/ma14030575

**Published:** 2021-01-26

**Authors:** Sanja Dimter, Miroslav Šimun, Martina Zagvozda, Tatjana Rukavina

**Affiliations:** 1 Faculty of Civil Engineering and Architecture Osijek, Josip Juraj Strossmayer University of Osijek, 31000 Osijek, Croatia; mzagvozda@gfos.hr; 2 Civil Engineering Department, Zagreb University of Applied Sciences, 10000 Zagreb, Croatia; msimun@tvz.hr; 3 Faculty of Civil Engineering, University of Zagreb, 10000 Zagreb, Croatia; rukavina@grad.hr

**Keywords:** wood ash (WA) by-product, hot mix asphalt, physical and mechanical properties, indirect tensile strength, resistance to action of water, sustainable road pavement design

## Abstract

Today, the road construction profession is more than ever facing limited and increasingly expensive resources for component materials of asphalt mixtures, which has also led to the need for continuous research on the use of waste materials. One such potentially usable waste material is ash obtained by the combustion of wood biomass that is used to produce heat and electricity. The goal of this paper is to ascertain the possibility of using wood ash (WA) as the filler in asphalt concrete mixtures for the base-wearing layers of a pavement. The properties of Marshall stability (MS), quotient (MQ) and deformations, and the indirect tensile strength of water-conditioned samples and dry samples were tested on asphalt samples of an AC16 surf mixture with different contents of wood ash as the filler. The obtained values of MS and MQ indicate that a 50% content of bio ash in the filler results in an increase in asphalt’s resistance to the appearance of plastic deformations and greater tensile strength and in good asphalt resistance to the action of water.

## 1. Introduction

The use of biomass as an energy source has a long tradition in Croatia [1], but it has been used on a small scale, primarily for heating houses and other structures and in food preparation. Today, prompted by ecological reasons and various European and national regulations, this renewable source is again being used to produce energy, but on a larger scale. In recent years, the obligations prescribed by the European Directive 2009/28/EC [2] to generate 20% of energy from renewable sources by 2020 has led to increased development of power plants that generate energy from biomass. According to data from the Croatian Energy Market Operator Ltd. [3], there are currently 35 biomass-fueled power plants with an installed power of 74,209.00 kW and another 15 power plants (35,599.00 kW) waiting to start operations.

Directive 2009/28/EC [2] defines biomass as “the biodegradable fraction of products, waste and residues from agriculture (including vegetal and animal substances), forestry and related industries including fishing and aquaculture, as well as the biodegradable fraction of industrial and municipal waste.” In thermal power plants that use biomass to generate heat and electricity, the combustion of biomass creates large quantities of ash (so-called bio ash).

According to A. Demirbas [4], biomass is considered one of the most diverse and valuable sources of renewable energy in the world. Biomass can be divided in wood biomass and herbaceous biomass. It is created by the action of humans and nature and includes wood, wood industry by-products, forestry waste, agricultural waste and waste from the food industry such as straw, waste from diverse grains, olive pomace, rice husks, sunflower husks, and date seeds. Ash is created during combustion from inorganic matter contained in the fuel through complex chemical and physical processes, and three different fractions of ash are produced: bottom ash, coarse (cyclonic) fly ash, and filter fly ash [5].

The particle size range and the granulometric composition of a particular fraction of biomass ash are the result of the technologies for separating and collecting the ash and the origin of the biomass that is combusted [6]. The physical and chemical properties of bio ash (BA) depend on the origin of the biomass and the part of the plant from which it is made, the quantity of mineral impurities, the location of the source of the biomass, the way in which the biomass is collected and processed, and the technology and temperature of combustion [7,8].

A review of the existing research shows that BA belongs to the group of waste materials that can be used in construction in their original form or with additional processing, depending on their origin and chemical and mineralogical composition [5,9].

The chemical composition of BA, especially the content of CaO and pozzolan, indicates the possibility of partial or complete replacement of traditional binders, cement, and lime during the stabilization of the subgrade materials with a lower load bearing capacity [10,11,12,13,14,15], in the base layers of pavement structures [16,17,18,19,20] or in concretes [21,22]. With the absence of binding properties, BA can be used as a replacement for a mineral filler or aggregate in asphalt mixtures, a filler in concretes, and as a replacement for smaller aggregate in base layers of pavement structures, depending on the fraction of ash.

BA is characterized as non-hazardous waste, and since large quantities of BA in landfills are expected, its reuse/recycling is strongly encouraged. Moreover, a wider interested group of individuals can take part in the mutual goal of finding use of waste material. BA is one of the alternative materials with great potential for use in construction, especially in road construction [9], where it can be used in all the pavement structure layers.

## 2. State of the Art

Asphalt mixtures are quality mixtures with a complex, highly elastic behavior. They are made of various stone aggregate fractions, sand, filler, bituminous binder, and, as required, other additives. They are used in the construction of the surface, binding, and base layers of an asphalt pavement structure. The composition and the components of asphalt mixtures are carefully selected to ensure a stable and durable surface resistant to the appearance of plastic deformations at higher temperatures, to water and damage at low temperatures, and roughness and skid resistance for driving safety. In addition to these requirements for constructing an asphalt layer, there is also a requirement for cost-efficiency in the procurement and the quality of the constituent materials.

Fly ash made by the combustion of coal in thermal power plants found their application in asphalt mixtures first [23,24,25,26]. Various applications of this type of ash were the most and longest researched. With time, the possible application of other types of ash were researched, such as ash from municipal waste incineration plants [27,28], from paper mills [29], and bio ash of various origins [30,31,32,33,34,35,36,37,38,39,40,41,42,43,44,45,46,47,48]. Compared to the properties of ash from the combustion of coal, the properties of BA have a much wider range, and it is often necessary to carry out certain preliminary actions to meet the requirements of a particular application set by technical specifications. This wide range of properties also points to the fact that it is impossible to determine an adequate application of a particular BA without detailed research. 

As part of an extensive multi-year project funded by the Italian Ministry of Agriculture, Food, and Forestry, a study was made of the potential of 27 different types of BA collected with 12 bioenergy plants as possible fillers in the production of asphalt mixtures [30]. The bio ashes had a mixed composition, consisting of ash from waste wood, bark, groundnut shells, rice husks, and straw. The filler is an integral component of asphalt mixtures, the content of which has a significant impact on the mechanical properties of the mixtures. The research included extensive tests of the physical and chemical properties of the BA as possible fillers. Since it was also necessary to determine possible harmful impacts on the environment for each material, a leaching test for pure BA (without a bituminous film) was carried out. The tests confirmed large variability of the composition for each type of BA, samples of which were collected at various time intervals (two times per year). In these tests, it was not possible to determine a clear connection between the type of biomass and the properties of the BA. Melotti et al. [30] concluded that most of the tested BA could be considered a potential replacement for a natural filler in asphalt mixtures that would not be hazardous for the environment, and that a complete answer would be obtained by testing the properties of the asphalt mixtures. 

Sargin at al. [31] researched the use of rice husk ash (RHA) as a mineral filler in a hot asphalt mixture. Four different series of asphalt concrete with limestone aggregate and with different amounts of the mineral filler (4%, 5%, 6%, and 7%) were produced. The optimum content of bitumen and the value of Marshall stability (MS) was determined for the samples using the standardized Marshall test. In further tests, a series of asphalt concrete mixtures with 5% of the filler was selected, because it had the highest stability with the limestone (LS) filler. The content of RHA in the filler was set at 0%, 25%, 50%, 75%, and 100%. Testing was carried out on the samples and an analysis of the Marshall stability (MS) results confirmed the possibility of the application of RHA as a quality supplement for mineral filler in asphalt concrete. An asphalt mixture with 50% of the filler from RHA showed the best results. 

A similar test with a filler from RHA in asphalt concrete mixtures for wearing layers was conducted by Al-Hdabi [32]. The goal of the research was to determine changes in the mechanical and volumetric properties of the asphalt mixture and the durability of the mixture in relation to the control asphalt mixture with a filler of Portland cement. The content of bitumen in asphalt mixtures was 5.5%. The results showed that the stability of the asphalt mixture with RHA according to Marshall was 65% greater than the stability of the control mixture. The same trend was also observed in the results of testing Marshall stiffness. The author explained the results by noting “that RHA non-spherical particles act as a reinforcing material to the asphalt cement and increase the cohesion and stiffness comprehensively.” The obtained results of testing the indirect tensile strength (ITS) of the mixture with a filler from RHA were 10% greater in comparison to the value of the results of the control asphalt mixture. Besides improving the mechanical properties of the asphalt mixture with RHA, improvements in their durability and water sensitivity were also noticed. 

Testing the possible application of two types of BA, from rice husks and date seeds, as the filler in hot asphalt mixtures was conducted by Tahami et al. [33]. The content of each type of BA in asphalt mixtures was 0% (the control mixture with the standard filler), 25%, 50%, 75%, and 100%. The mixtures were tested for their mechanical properties, and the results were compared with the control asphalt mixture with a mineral filler. The asphalt mixtures with RHA and date seed ash showed greater stability and stiffness modulus compared to the control mixture with standard filler. The content of BA in the mixtures improved thermal sensitivity of the asphalt mixture and adhesion between bitumen and the aggregate. It also increased the resistance of the mixture to wheel tracking and fatigue.

The impact of wood bio ash (WA) as a replacement for part of the filler in asphalt mixtures was researched by Bi and Jakarni [34]. The content of WA in the total mass of the filler was 25%, 50%, 75%, and 100%. The results showed that the asphalt mixture with wood ash as part of the filler had a greater value of the resilient modulus (MR) (as much as 40%) compared to the control mixture with the mineral filler. They also showed that the resistance to fatigue was increased, and permanent deformation of the asphalt mixture was reduced. It was observed that the stability of the asphalt mixture decreased as the content of WA in the filler increased, but those reduced values of stability still met the criteria prescribed by the Technical Conditions for Road Works (Standard Specification for Road Work, Kuala Lumpur: JKR). 

The impact of a filler of wood sawdust ash (WSA) and RHA on the properties of the asphalt mixture was researched by Boura and Hesami [35]. The content of bio ash in the limestone filler was 0%, 25%, 50%, 75%, and 100%. The test results showed that the mixture with 75% RHA and 25% limestone filler had greater stability according to Marshall and a lower water sensitivity compared to the control mixture with a 100% limestone filler, while the asphalt mixture with 25% of WA showed better tensile properties than the control. The results of the resilient modulus of the mixtures with bio ash were lower than the results for the control mixture. With the results of testing Marshall stability, indirect tensile strength, and moisture sensitivity, the authors concluded that the optimum content in the filler was 75% for RHA and 25% for WSA.

In addition to the use of BA as an additive to, or a complete replacement of, the mineral filler, the possibility of its use in the modification of the bituminous binder was researched. In research by Xue at al. [36], two types of ash from biomass were used to modify bituminous binders: RHA and WSA. The dynamic rheological behavior of bitumen, high temperature sensitivity, and the aging of bitumen were tested. The results showed that there was no chemical reaction between the bituminous binder and BA. Comparing the two types of bio ash, RHA demonstrated a better effect in improving the physical properties of the modified bituminous binder. Stability tests at high temperatures showed that bitumen was stable when the content of BA (regardless of the type) was less than 20%. It was also determined that both types of BA improved resistance to rutting.

Arabani and Esmaaeli [37] researched the impact of bio ash from groundnut shells (groundnut shell ash—GSA) as a modifier of the binder on the properties of the hot asphalt mixture (HMA). Bio ash was added in quantities of 5%, 10%, 15%, and 20% of the total mass of bitumen, and all the prescribed tests were carried out on the asphalt mixtures. The results showed that a binder with a content of GSA of up to 10% remained stable at high temperatures. Adding GSA to bitumen resulted in an increase of the softening point, viscosity, and resistance to wheel tracking, and in a reduction of ductility and resistance to damage from fatigue and low temperatures. It proved that modification of bitumen with bio ash from groundnut shells improved the mechanical properties of the asphalt mixture.

The above mentioned research of various types of BA confirmed its potential for use in asphalt mixtures, and the application of BA cannot only reduce its quantity at landfills, but it can also improve the physical and mechanical properties and durability of the asphalt mixture. Of course, the variability of the composition of BA should always be considered, and its application should be confirmed by conducting full laboratory tests. 

## 3. Objective of This Study

As mentioned, the development and increase in the number of biomass power plants led to the production of increasing quantities of BA in Croatia. Wood ash, used in this study, is a by-product from a company in eastern Croatia engaged in wood processing and the production of parquet and other wood products. Since 2011, it has also operated a co-generation plant for the production of electricity and heat based on the combustion of wood biomass, which uses a boiler with combustion technology on a sloping grate where combustion temperatures can reach about 660 °C [38]. To produce heat and electricity, co-generation uses the remains from the sawmill, but it can also include wood bark (but not sawdust) and chipped wood. About 110 t of the wood biomass are combusted daily, which represents an annual consumption of about 40,000 tons of wood biomass. In addition to energy, wood ash is also produced during combustion, in an amount of approximately 4% of the combusted biomass, or about 3–4 tons per day [38]. The quantities of ash produced vary depending on the quality of the chipped wood and other combusted raw materials. Based on the place of their creation and accumulation, ashes are divided in three fractions: bottom ash that is collected under the boiler grate, and fly ash collected on a cyclone de-duster and electrostatic filter. The daily quantities of ash that are produced are significant. There is currently no systematic method for recovering the ash. It is mostly deposited, but it is also used to backfill local access routes and in small quantities as a soil fertilizer in orchards [38].

The goal of this research is to examine the physical and mechanical properties of asphalt mixtures of asphalt concrete type AC16 surf with different contents of WA in the filler and determination of the water sensitivity of bituminous specimens. The expected application of the results would be a more rational and cost-efficient design of mixtures intended for use as asphalt layers of pavement structures.

## 4. Materials 

Laboratory tests were carried out on the asphalt mixture of asphalt concrete type AC 16 surf, which is used for the construction of wearing and base-wearing layers [39], in accordance with HRN EN13108-1 [40], with a binder with a content of 4.9% of road construction bitumen, type B50/70, with properties according to HRN EN 12591 [41]. 

### 4.1. Aggregates

The properties of crushed coarse and fine aggregate used in the asphalt mixture are shown in Table 1. An aggregate with a carbonate composition was used. The properties of the aggregate were determined according to the harmonized standard HRN EN 13043 [42]. 

**Figure 1:**
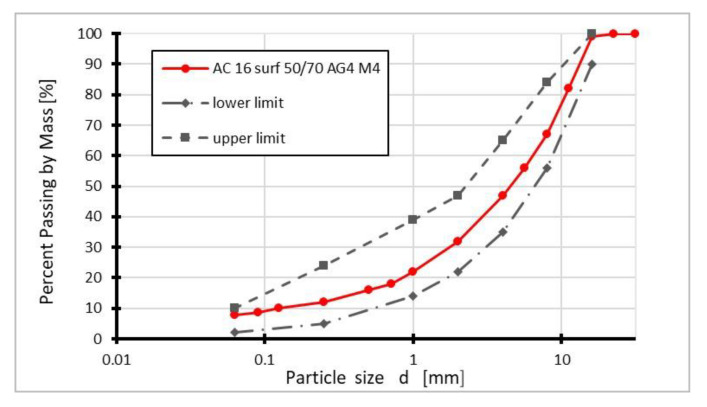
Granulometric composition of the stone mixture for preparing AC 16 surf.

The granulometric composition of the stone mixture for preparing the AC 16 surf asphalt concrete mixture is presented in Figure 1.

### 4.2. Filler

A mineral filler (industrial) and a filler from wood ash were used as the basic fillers in different proportions in the AC 16 surf asphalt mixture. The properties of the mineral filler and the filler from WA were determined according to HRN EN 13043 [42].

**Figure 2:**
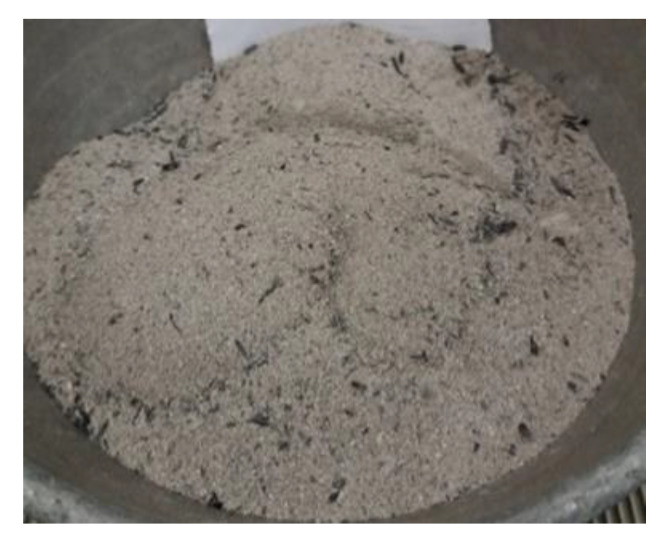
Cyclone ash from wood biomass.

Mineral filler (industrial) is of carbonate composition (limestone and dolomite) of sedimentary origin. As a supplement to the mineral filler, WA was used. It is a cyclone ash, i.e., fly ash collected on a cyclone de-duster (Figure 2). 

The chemical composition of the wood ash is shown by the portion of individual components (mass %): MgO = 3.06, Al_2_O_3_ = 0.44, SiO_2_ = 4.05, P_2_O_5_ = 2.90, SO_3_ = 1.59, K_2_O = 2.82, CaO = 46.91. As seen from the composition, the main component is calcium oxide, which can increase adhesion between bitumen and aggregate and, thus, decrease the aggregate nakedness. The granulometric composition of the WA filler can be seen in Table 2. The results show that the granulometric composition of the tested WA met the requirements set at the upper limit sieve (2 mm), while it was somewhat below the prescribed range at the lower (0.063 mm) and middle (0.125 mm) limit sieve.

**Figure 3:**
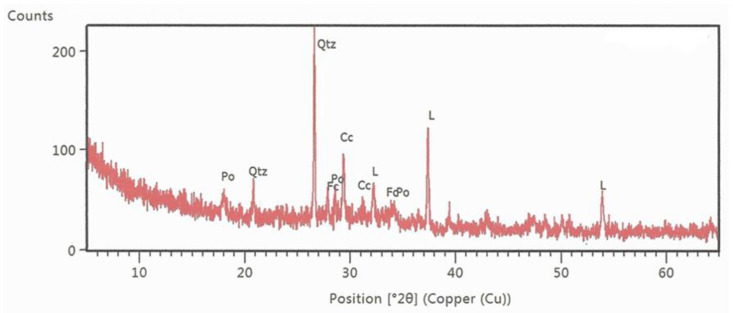
The results of the XRD analysis of wood ash [43].

Using X-ray diffraction analysis (XRD), it was determined that the main components of wood ash were calcite, quartz, and CaO, and, in smaller quantities, portlandite (Ca(OH)_2_) and fairchildite (K_2_Ca(CO_3_)_2_) [43]. Figure 3 presents the results of the XRD analysis of cyclone ash.

**Figure 4:**
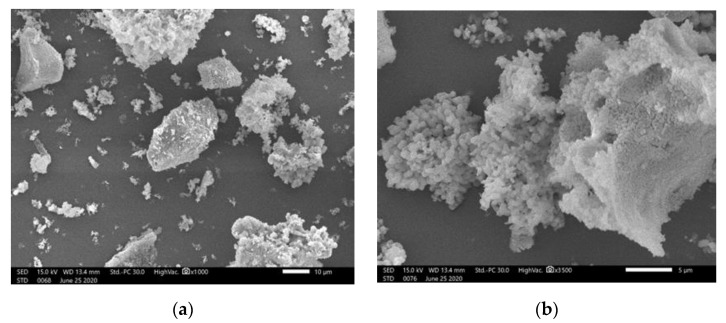
SEM microphotograph of wood ash particles: (**a**) magnification SEM_MAG = 1000×; (**b**) magnification SEM_MAG = 3500×.

For better understanding the effect of wood ash on asphalt mixtures properties, its morphology is important. Therefore, scanning electron microscopy (JEOL SEM JSM-IT200) was used for images of ash particles shape and size (Figure 4) The SEM images showed that the wood ash was composed of particles with different shapes and sizes. The ash particles were irregular in shape and had rough, porous surfaces; the particle sizes were also very uneven. The overall structure of particles did not follow a specific pattern.

### 4.3. Asphalt Binder

Bitumen type B50/70 was used as the binder in the asphalt mixture. It had the properties set in HRN EN 12591 [41]. The content of bitumen in the mixture was 4.9%, and its density was 1.020 Mg/m^3^.

### 4.4. Composition of the Asphalt Mixtures

Regarding the different compositions of the filler, four mixtures of asphalt concrete AC 16 surf were designed, so that the initial filler consisted only of the mineral filler. It was then changed in increments of 25% as follows:

- A mixture with 100% of the mineral filler (control mixture)
- B mixture with 25% WA, 75% mineral filler
- C mixture with 50% WA, 50% mineral filler
- D mixture with 75% WA, 25% mineral filler

## 5. Test Methods

Before conducting testing of the asphalt mixtures properties, it was necessary to determine if the WA had the required properties for the filler, and if it would have an impact on the environment.

### 5.1. Methods for Testing the Release of Hazardous Substances from WA

To determine the environmental impact of WA waste material and to identify hazardous substances release, testing of the eluate was carried out. The characteristics of the dry matter was tested at 105 °C in accordance with the standard HRN ISO 11465 [44], and the eluate was prepared according to HRN EN 12457-4 [45]. The concentration of hydrogen ions (pH) was also tested, as were the content of fluorides, sulphates, chlorides, the phenolic index, and the contents of the total dissolved solids (TDS) and dissolved organic oxide (DOC). According to the standard HRN EN ISO 17294-2 [46], the content of the following dry matter was determined: arsenic (As), barium (Ba), cadmium (Cd), total chromium (Cr), copper (Cu), mercury (Hg), molybdenum (Mo), nickel (Ni), lead (Pb), antimony (Sb), selenium (Se), and zinc (Zn). The concentration of hydrogen ions in water (pH in water) of BTEX (benzene, ethylbenzene, toluene, and xylenes), of total organic oxide (TOC), total hydrocarbons (mineral oils C10 to C40), PCBs (polychlorinated biphenyls), and the sum of PAHs (polycyclic aromatic hydrocarbons) were tested separately. The properties and quality of fine particles were tested according to the standard HRN EN 933-9:2013 [47]. Water solubility of the filler was tested according to the standard HRN EN 1744-1:2012 Item 16 [48]. 

### 5.2. Methods for Testing WA as a Filler

In addition to the basic condition that enables the application of wood ash as a filler (the granulometric composition of WA (Table 2)), its other properties were also tested. The density of the filler was tested according to HRN EN 1097-7 [49] at a temperature of 25 °C and with a water density of 0.99704 Mg/m^3^. The proportion of voids in the dry compacted filler according to Rigden was tested (HRN EN 1097-4 [50]) with four guides of the Rigden device.

Regarding other properties of WA as a filler, the following items were tested:

- difference of softening points ΔR&B according to HRN EN 13179-1;
- sensitivity of the filler to water according to HRN EN 1744-4; and
- bitumen number according to HRN EN 13179-2.

### 5.3. Testing the Physical and Mechanical Properties of Asphalt Mixtures

In accordance with the standard HRN EN 12697-35 [51], four bituminous mixtures were mixed at 160 °C for 3 min with 0% (control), 25%, 50%, and 75% content of a WA filler. Asphalt specimens were prepared by an impact compactor according to HRN EN 12697-30 [52] at 150 °C, with a compaction energy of 2 × 50 strokes. The physical and mechanical properties tested in accordance with the standard HRN EN 12697-8 [53] on bituminous mixtures with different contents of WA in the filler are presented in Section 6.3.

The mechanical properties of the asphalt mixture were evaluated by Marshall stability (MS) and Marshall flow and indirect tensile strength of water-conditioned samples and dry samples.

For the Marshall asphalt samples, the stability and deformations were tested (HRN EN 12697-34 [54]). On the basis of the relationship of the stability (kN) and deformations (mm), the stiffness (kN/mm), i.e., Marshall quotient, was calculated.

### 5.4. Testing the Sensitivity of Asphalt Samples to Water

Water sensitivity is one of the main causes of asphalt damage and deterioration. It is characterized by the loss of the adhesive bond between the bituminous binder and the aggregate and the weakening of the cohesive bonds in the bituminous binder itself due to the action of the traffic load and the presence of moisture. 

To test water sensitivity, asphalt specimens were prepared using an impact compactor according to HRN EN 12697-30 [52] at 150 °C, with a compaction energy of 2 × 35 strokes. Then, according to the standard HRN EN 12697-12 [55], testing of the asphalt samples was performed with procedure A at 15 °C. Testing was conducted on a group of four dry samples and a group of four water conditioned samples, before and after vacuuming, for all types of asphalt mixtures (4 × 2 × 4 samples in all). The water conditioning temperature was 40 °C, and conditioning time was 72 h. On 32 Marshall samples, with all the asphalt mixtures (A, B, C, D), the dimensions were determined according to HRN EN 12697-29 [56], specifically, the heights ranging from 63.4 to 65 mm and diameters ranging from 101.5 to 101.6 mm. The density was determined according to the standard HRN EN 12697-6 [57]. 

The tensile strengths of the asphalt samples were determined by the indirect tensile strength (ITS) method, in accordance with the standard HRN EN 12697-23 [58]. 

## 6. Results and Discussion

### 6.1. The Results of Hazardous Substance Release from WA

After testing the properties of hazardous substance release from WA, three values (sulphates, the content of total dissolved solids (TDS) and total chromium) were above the maximum allowed concentrations (MAC) according to [59,60,61]. The results are presented in Table 3. Based on these results, this construction product cannot be used for embedding in an embankment and backfilling ditches. However, since WA in the bituminous mixture was encased in a bituminous binder, it was concluded that the hazard was sufficiently annulled for this phase of the research. A similar conclusion regarding to the impact of bioash on the environment, was pointed out by Melotti et al. (2013) [30], who investigated 27 types of bioash used as a filler in asphalt mixtures. The leaching test results indicated that the use of bioash in asphalt mixes is safe for the environment because of binder coating on the filler particles. 

When testing the quality of fine particles and the solubility of the filler in water, satisfactory properties of WA as the filler were observed.

### 6.2. The Results of Testing WA as an Added Filler

The density of the added filler according to the standard HRN EN 13043 [42] was not prescribed, and the density value of 2.689 Mg/m^3^ was observed. The value of the content of voids in the dry compacted filler was 56% and was above the upper prescribed limit (V38/45), which indicated that a greater amount of bitumen would be necessary to form the mortar of the asphalt mixture if bio ash was to be used, i.e., the time of mixing and homogenizing would have to be extended. 

The test determined a difference in the softening points ΔR&B > 25 °C and, according to HRN EN 13043:2003/AC:2006, it was in class ΔR&B25, while the observed bitumen number was 59, placing it in class BN53/62.

The testing procedure confirmed that the content of water in WA as the filler was <1% (m/m), and that wood ash as the filler in bituminous mortar had no water sensitivity because there was no water turbidity.

### 6.3. The Impact of WA on the Volumetric Properties of the Asphalt Mixture 

**Figure 5:**
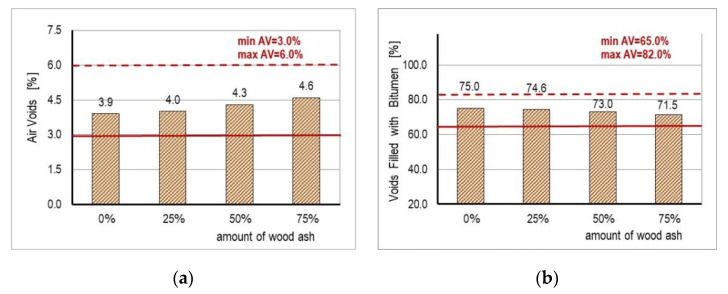
(**a**) Air void results; (**b**) voids filled with bitumen results.

**Figure 6:**
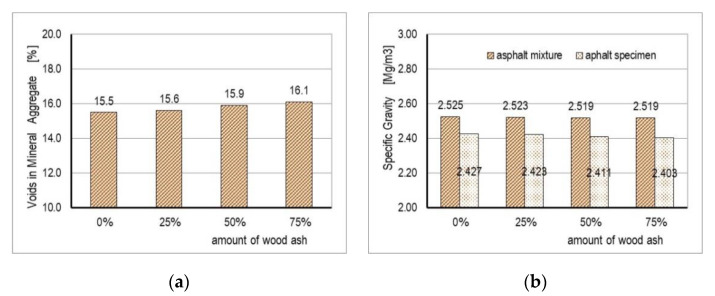
(**a**) Voids in mineral aggregate results; (**b**) specific gravity results.

The results of testing the physical and mechanical properties of the mixture are shown in Table 4 and Figure 5 and Figure 6.

The physical characteristic that is most frequently connected with the behavior of the asphalt mixture is the content of air voids in the mixture. Research has shown that the content of air voids affects the stability and durability of the asphalt mixture. If minimum values of the content of voids (below 3%) are detected, the appearance of rutting can be expected, while if the values are above 8%, due to the accelerated oxidation of the bituminous binder, there is a danger of the premature appearance of cracks and crumbling [62]. According to the Croatian General Technical Requirements for Road Work (CGTC) [63], for wearing layers and heavy traffic load, the values of air voids in the asphalt mixture are 3–6%.

The content of voids filled with bitumen is inversely proportional to the content of the air voids. When the content of voids filled with bitumen exceeds the limit of 82–85%, the asphalt mixture becomes unstable, and the appearance of rutting can be expected. According to [63], for surface layers and heavy traffic, the values of voids filled with bitumen are 65–82%.

From the obtained results, it is evident that the content of voids and the content of voids filled with bitumen of all of the mixtures met the CGTC [63]. The content of voids in the mixture increased evenly as the content of WA in the binder increased, from 3.9% (control mixture) to 4.6%, which was also the highest amount noticed in mixture D. The content of voids filled with bitumen decreased with the increase of WA in the filler, from 75% (control mixture) to 71.5% for mixture D with the highest content of WA.

The content of voids in the aggregate depends on its granulometric composition and on the form and texture of the aggregate surface. For the tested mixtures, the content of voids ranged from 15.5% (control mixture) to 16.1% (mixture D), increasing with the increase of the content of WA in the filler. 

The density of the mixture was reduced with an increase of the content of WA in the filler, i.e., with an increase of the content of air voids. With an increasing amount of WA in the filler, mastic viscosity increased. Decreasing of density and increasing of air voids for mixtures with WA in comparison with the control mixture A, may have been because the stiffness of bitumen increased when adding WA. A similar conclusion was stated by Al-Hdabi [32], who investigated properties of asphalt concrete mixture with Rice Hush Ash as a filler.

### 6.4. The Impact of WA on the Results of the Stability and Stiffness According to Marshall 

**Figure 7:**
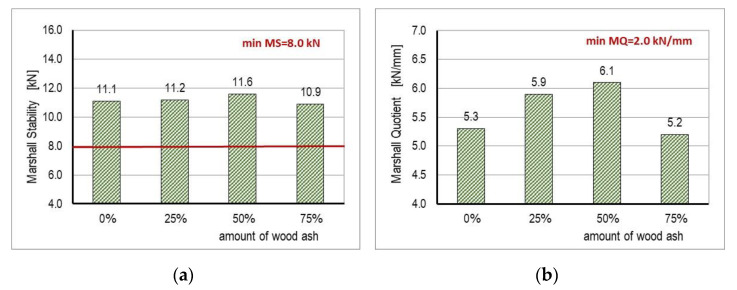
(**a**) Marshall stability results; (**b**) Marshall quotient results (stiffness).

The values of Marshall stability (MS) determined by the standard HRN EN 12697-34 [54] are shown in Figure 7a. They showed an increase in stability values (up to 5%) relative to control mixture A for mixtures B and C. The stability value was the highest for mixture C, with a 50% content of WA in the filler, and was 11.6 kN. For mixture D, with 75% WA, the MS value was lower than the value of the control mixture and was 10.9 kN. The obtained results of Marshall stability can be explained as follows: At lower percentages, the fine-grained composition of WA allowed it to fill in the empty spaces in the binder and improved mastic performance and Marshall stability (mixtures B and C). At higher percentages of WA (mixture D), the round corners and small particles of WA created a flat surface by filling the surface cracks of the aggregates. Therefore, the fastening between the aggregates decreased and causes lower Marshall stability of asphalt mixtures. The above results are consistent with the results obtained by other investigations described in [31,32,33,35]. In addition, the stiffness and adhesion strength of mastic is an important factor in resistance against low temperature cracking due to bitumen oxidation, fatigue cracking, and the appearance of lower resistance on rutting.

Since the minimum value of stability of an asphalt mixture for wearing layers and heavy traffic load, according to CGTC [63], is 8.0 kN and with a reduced stability value compared to the other tested mixtures, mixture D met the required stability criteria for application in the asphalt layer.

Figure 7b shows the values obtained for stiffness (Marshall quotient—MQ). A trend similar to the stability results could be noticed. The greater stability value divided by deformation indicated a mixture with greater stiffness, which means that the mixture was more resistant to permanent deformations. The results show that with increased content of WA in the filler, the MQ values also increased. Mixture C had the highest MQ value with 50% of WA content. With an increase in the WA content (mixture D), the value decreased. According to CGTC [63], the minimum value is MQ = 2.0 kN/mm. 

**Figure 8:**
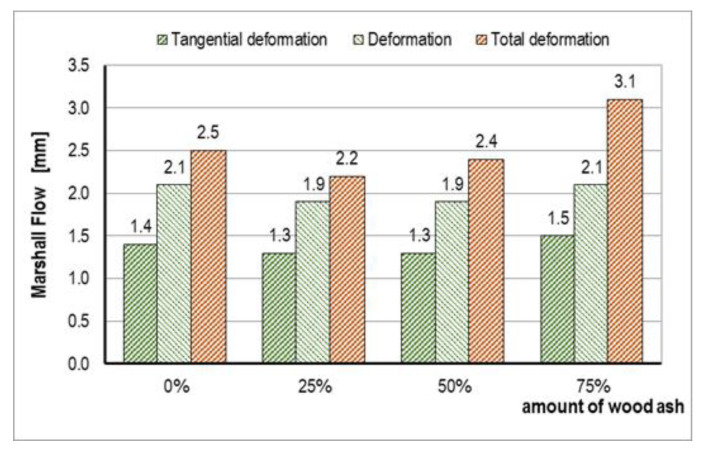
Marshall flow results.

In accordance with HRN EN 12697-34 [54], the deformations, tangential deformations, and total deformations were determined (Figure 8). The mixtures with 25% and 50% of WA had less deformations compared to the control mixture A. With a further increase in the content of WA, the deformations increased to the maximum value of 3.1 mm.

### 6.5. The Impact of WA on the Results of Indirect Tensile Strength—ITS

**Figure 9:**
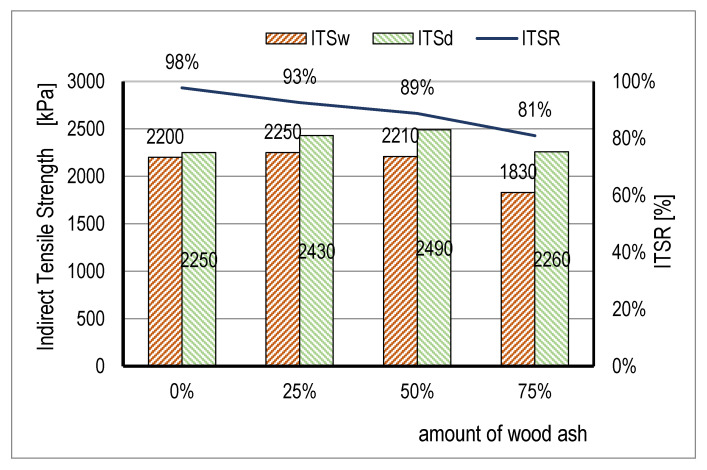
Results of indirect tensile strength (ITS) and ratio (ITSR).

The values of the indirect tensile strength of water conditioned samples (ITSw) and dry (ITSd) samples are presented in Figure 9. The tensile strength results of the water conditioned samples (ITSw) for mixtures B and C were slightly higher (up to 2%) compared to the tensile strength result of control mixture A. The samples of mixture B, with a content of 25% WA, had the highest tensile strength (2250 kPa). The samples of mixture D, with a 75% WA content, had a lower tensile strength than the samples of the control mixture A by approx. 20%.

A similar trend for the increase of the tensile strength values compared to control mixture A was also noticed in the dry samples (ITSd) for all the mixtures. The samples of mixture C, with a 50% content of WA, had the highest tensile strength value (2490 kPa). The samples of mixture D, with 75% WA, had a tensile strength approximately 10% lower compared to mixture C, and a slightly greater stiffness compared to control mixture A. Generally, the round corner particles of WA improved asphalt mixtures tensile performance. They also had a tendency to absorb bitumen, therefore, by increasing the amount of WA, the effective bitumen content and tensile strength decreases. The obtained results confirmed the conclusion of Boura and Hesami [35], who investigated wood saw ash (WSA) as a filler; the best tensile performance had asphalt mixtures with 25% WSA and 75% limestone filler.

The results of the indirect tensile strength ratio (ITSR) of the water conditioned (ITSw) samples and of dry (ITSd) samples were an indicator of the sensitivity of asphalt samples to water. The values ranged from 81% to 98% in all of the tested samples (Figure 9), which confirmed good resistance of the asphalt samples to the effect of water. With increasing amounts of WA filler, mastic viscosity increased, and it increased the percentage of voids in the asphalt specimen.

Therefore, compared to the samples of control mixture A, all three mixtures with a content of WA had a lower ITSR value. It can be concluded that the increase of the content of WA in the filler results in a reduced ITSR, i.e., that the mixtures with a higher content of WA are less resistant to water, but within the required values of resistance to the effects of water.

## 7. Conclusions

This research examined the impacts of wood ash (WA) as an additive to the filler on the physical and mechanical properties of the AC 16 surf asphalt mixture and the impact on the resistance of asphalt to the actions of water. An asphalt mixture of asphalt concrete type AC 16 surf consists of an aggregate with a carbonate composition and filler, which are bound with 4.9% of road construction bitumen. A mineral filler (industrial) is of carbonate composition (limestone and dolomite) of sedimentary origin. In the asphalt mixture were used both mineral filler and a filler from wood ash (cyclone ash) in different proportions. This research confirmed the usability of WA and its optimum content in the filler of asphalt mixtures and can be summarized as follows:

1. The results of the tested properties of WA as an added filler in asphalt mixtures confirm that there are no major deviations from the values for the mineral stone fillers prescribed by the standard. Due to the observed hazardous substances (Table 3), WA cannot be deposited at landfills as its own internal waste on its own or in a mixture with other internal waste materials with similar properties. However, bound with bitumen and built in an asphalt layer in a pavement structure, it is a part of the structure and should not be harmful to the environment.
2. The results of Marshall stability (MS) and Marshall quotient (MQ) showed that with increased content of WA in the filler up to 50%, the MS and MQ values also increase. Marshall stability (MS) increased by 4.5% (from 11.1 kN to 11.6 kN). The greater stability value divided by deformation indicates a mixture with greater stiffness and an Marshall quotient (MQ) increase by 15.0% (from 5.3 kN/mm to 6.1 kN/mm). The obtained results of MS and MQ had a direct effect on the greater resistance of asphalt to rutting under traffic load. For mixture D, with 75% WA, the MS (10.9 kN) and MQ (5.2 kN/mm) values were lower than the values of the other asphalt mixtures, but even with this reduced stability and stiffness, mixture D met the required Croatian technical criteria for application in the asphalt layer.
3. The tensile strength results of dry samples (ITSd) showed that with an increased content of WA in the filler up to 50%, ITSd results increased by 10.7% (from 2.250 kPa to 2.490 kPa). This result confirms a lower risk of cracks and a longer period of asphalt durability in exploitative conditions, respectively, better resistance to the degree of material fatigue. The samples of mixture D with a 75% WA content had a lower tensile strength than the other asphalt mixtures with WA (ITSd =2.260 kPa) but still were better than control mixture without WA (ITSd =2.250 kPa).
4. The indirect tensile strengths ratio results (ITSR = ITSw/ITSd) confirmed good resistance of the asphalt samples to the effect of water. The values ranged from 81% to 98% in all of the tested samples. All three of the mixtures with WA had a lower ITSR value than the control mixture without WA, which indicates that the increase of WA content in the filler resulted in a reduction of ITSR (93%, 89%, 81%). Since mixtures with a higher content of WA are less resistant to water, the optimum content of WA in the filler needs to be limited to 50%.
5. Although an increase in the content of voids in the mixtures (3.9%, 4.0%, 4.3%, 4.6%) was noticed with the increase of the content of WA (0%, 25%, 50%, 75%), the three mixtures with WA meet the criteria prescribed by Croatian technical criteria and, according to their physical and mechanical properties, they could be used in construction of asphalt pavements. However, according the ITRS results, the optimum content of WA in the filler needs to be limited to 50%.
6. This research confirmed the possibility of using cyclonic wood ash as a resource in the form of a constituent material of asphalt mixtures. Usage of wood ash can give economic and ecological benefits, in full accordance with the guidelines of sustainable development:
  (a) Usage of WA reduces the number of landfills and quantity of waste in landfills. About 110 t of wood biomass are combusted daily, which represents an annual consumption of about 40,000 tons of wood biomass. Wood ash is also produced during combustion, in an amount of approximately 4% of the combusted biomass, or about 3–4 tons per day. The daily quantities of produced ash are significant. Currently, there is no systematic method for recovering the ash; it is mostly deposited in landfills. Depositing in landfills is demanding and expensive and takes up valuable space. Given that the WA is characterized as non-hazardous waste and that large amounts of WA are expected in landfills, its reuse/recycling is strongly encouraged.
  (b) Usage of WA reduces the construction costs of pavement structures. The cost of asphalt mixture with up to 50% wood ash in a filler is 2–4% less than the cost of asphalt mixtures with a mineral filler (industrial). Given that asphalt mixtures are mixtures whose price significantly increases each project’s cost, even the smallest cost savings is desirable and welcomed.
  (c) Usage of WA reduces the need for natural aggregates and protects the environment. Savings in the cost of industrial filler production and protection of limited quantities of natural limestone and dolomite resources are achieved.

The presented results and conclusions are a product of research conducted on asphalt mixtures produced according to the European norms and Croatian technical regulations from local aggregate and filler partially replaced by locally generated wood ash. The paper points to some trends in properties of asphalt mixtures containing WA as a partial replacement of natural filler. It could be concluded that WA has the potential to be used as a partial substitute of industrial filler and to improve, to a certain degree, certain asphalt properties. In the end, it should be mentioned that the variability of the WA composition should always be considered and the possibility of application confirmed with full laboratory tests.

## Figures and Tables

**Table 1:** Physical properties of the aggregate.

Properties of Coarse Aggregate	Method	Value
Crushing resistance by “Los Angeles” method	HRN EN 1097-2	LA_30_
Surface abrasion resistance	HRN EN 1097-8 Additive A	AAV_20_
Wear resistance	HRN EN 1097-1	M_DE20_
Resistance to polishability	HRN EN 1097-8	PSV_30_
Resistance to freezing and thawing	HRN EN 933-6	WA_24_2
**Properties of Fine Aggregate**	**Method**	**Value**
Content of fine particles	HRN EN 933-1	F_10_
Quality of fine particles	HRN EN 933-9	MB_F_10

**Table 2:** Assessment of fine particles—classification of the filler according to HRN EN 933-10:2009.

Sieve Opening Size (mm)	Cumulative Passing (%)
2.0	100
0.125	78
0.063	49

**Table 3:** Hazardous substance release above permitted values.

Characteristic	Unit	Test Result	Maximum Allowed Concentration (MAC)
Sulphates	mg/kg DM	12.900	1.000
Content of total dissolved solids (TDS)	mg/kg DM	53.670	4.000
Total chromium (Cr)	mg/kg DM	1.95	0.5

**Table 4:** Physical and mechanical properties of bituminous mixtures.

Designation of Mixture	Content of WA in Filler (%)	Content of Voids (%)	Filling of Voids with Bitumen (%)	Voids in Stone Material (%)	Density of Asphalt Mixture (Mg/m^3^)	Density of Asphalt Sample (Mg/m^3^)
A	0	3.9	75.0	15.5	2.525	2.427
B	25	4.0	74.6	15.6	2.523	2.423
C	50	4.3	73.0	15.9	2.519	2.411
D	75	4.6	71.5	16.1	2.519	2.403

## Data Availability

The data presented in this study are available on request from the corresponding author.

## Abbreviations

Wood ash (WA), bio ash (BA), rice husk ash (RHA), wood sawdust ash (WSA), groundnut shell ash (GSA), limestone (LS), X-ray diffraction analysis (XRD), scanning electron microscopy (SEM), total dissolved solids (TDS), maximum allowed concentrations (MAC), hot asphalt mixture (HMA), Marshall stability (MS), Marshall quotient (MQ), indirect tensile strength (ITS), indirect tensile strength of water conditioned samples (ITSw), indirect tensile strength of dry samples (ITSd), indirect tensile strength ratio (ITSR), Croatian General Technical Requirements for Road Work (CGTC).
